# The History, Diagnosis and Treatment of the Fevers of the United States

**Published:** 1852-11

**Authors:** 


*The History, Diagnosis, and Treatment of the Fevers of the United States.* By ELISHA BARTLETT, M. D., Professor of Materia Medica and Medical Jurisprudence in the College of Physicians and Surgeons of the University of the State of New York; member of the American Academy of Arts and Sciences; Author of an Essay on the Philosophy of Medical Science, &c. &c. Third edition, revised. Philadelphia; Blanchard and Lea. 1852.

*Clinical Reports on Continued Fever, based on Analyses of one hundred and sixty-four cases; with remarks on the Management of Continued Fever; the identity of Typhus and Typhoid Fever; Relapsing Fever*; *Diagnosis, &c. To which is added a Memoir on the Transportation and Diffusion by Contagion, of Typhoid Fever, as exemplified in the occurrence of the disease at North Boston, Erie Co., N. Y.* By AUSTIN FLINT, M. D., Professor of the Principles and Practice of Medicine, and of Clinical Medicine, in the University of Buffalo, and Editor of the Buffalo Medical Journal. Buffalo: Geo. H. Derby & Co. New York: Geo. P. Putnam, 10 Park Place. Chicago: D. B. Cooke & Co. 1852.

This third edition of Dr. Bartlett's excellent treatise on the fevers of the U. States brings the subject down to our own time. Commencing with typhoid fever, the perpetual continued fever of the United States and of most temperate climates, he enters fully into its symptoms and mode of treatment. The symptoms of typhoid fever have been as well studied as those of any other disease, and are well detailed by Dr. Bartlett, and indeed have been so carefully described by Dr. Louis, that subsequent writers have had comparatively little to do but to repeat his graphic delineations of the disease. The method of treating it the author gives in extenso, including under separate heads the modes pursued by the different physicians who have written special treatises upon the disease.

Typhus fever is also described at large and is admitted by the author to be a disease different in character from typhoid fever, the absence of the characteristic lesions of the latter disease as well as the difference in symptoms establishing the diagnosis between them. The two diseases at times, however, approach each other very nearly and are closely allied ; perhaps they are sometimes found united, that is, the same patient on some rare occasions has some of the symptoms of typhus and some of those of typhoid fever.

Dr. Bartlett next proceeds to the subject of periodical fevers, including intermittent, remittent, and congestive fevers ; these are well described and the modes of treating them accurately pointed out. He finishes his work with yellow fever, which of course he is not able to delineate from his own observation, but he has given a very good analysis of the symptoms drawn from the histories of the disease published by different observers.

The reports by Dr. Flint of Buffalo include many points of interest to be added to the history of these diseases. It is of course a less perfect treatise than that of Dr. Bartlett, but contains many useful facts produced from his own experience. It forms an interesting volume for those who are engaged in studying these diseases, although not affording a complete history of them. Amongst the series of detached essays it contains, is one on the contagion of a set of cases of typhoid fever, which he considers to be very clearly shown. In our own experience, we have never seen any facts of the same character, but they have been so often stated by different observers, that we have no doubt that the disease may be sometimes, although very rarely, contagious.

